# The Endocannabinoid System: A Potential Target for the Treatment of Various Diseases

**DOI:** 10.3390/ijms22179472

**Published:** 2021-08-31

**Authors:** Henry Lowe, Ngeh Toyang, Blair Steele, Joseph Bryant, Wilfred Ngwa

**Affiliations:** 1 Biotech R & D Institute, University of the West Indies, Mona 99999, Jamaica; lowebiotech@gmail.com (H.L.); jbryant@ihv.umaryland.edu (J.B.); 2 Vilotos Pharmaceuticals Inc., Baltimore, MD 21202, USA; ngeh.toyang@flavocure.com; 3 Flavocure Biotech Inc., Baltimore, MD 21202, USA; 4 Department of Medicine, University of Maryland Medical School, Baltimore, MD 21202, USA; 5 Brigham and Women’s Hospital, Dana-Farber Cancer Institute, Harvard Medical School, Boston, MA 02215, USA; wngwa@bwh.harvard.edu; 6 Johns Hopkins University School of Medicine, Baltimore, MD 21218, USA

**Keywords:** *Cannabis sativa* L., endocannabinoid system, cancer, anxiety, depression, cannabinoids, phytocannabinoids, endocannabinoids

## Abstract

The Endocannabinoid System (ECS) is primarily responsible for maintaining homeostasis, a balance in internal environment (temperature, mood, and immune system) and energy input and output in living, biological systems. In addition to regulating physiological processes, the ECS directly influences anxiety, feeding behaviour/appetite, emotional behaviour, depression, nervous functions, neurogenesis, neuroprotection, reward, cognition, learning, memory, pain sensation, fertility, pregnancy, and pre-and post-natal development. The ECS is also involved in several pathophysiological diseases such as cancer, cardiovascular diseases, and neurodegenerative diseases. In recent years, genetic and pharmacological manipulation of the ECS has gained significant interest in medicine, research, and drug discovery and development. The distribution of the components of the ECS system throughout the body, and the physiological/pathophysiological role of the ECS-signalling pathways in many diseases, all offer promising opportunities for the development of novel cannabinergic, cannabimimetic, and cannabinoid-based therapeutic drugs that genetically or pharmacologically modulate the ECS via inhibition of metabolic pathways and/or agonism or antagonism of the receptors of the ECS. This modulation results in the differential expression/activity of the components of the ECS that may be beneficial in the treatment of a number of diseases. This manuscript in-depth review will investigate the potential of the ECS in the treatment of various diseases, and to put forth the suggestion that many of these secondary metabolites of *Cannabis sativa* L. (hereafter referred to as “*C. sativa* L.” or “medical cannabis”), may also have potential as lead compounds in the development of cannabinoid-based pharmaceuticals for a variety of diseases.

## 1. Introduction

### 1.1. History 

The Endocannabinoid System (ECS) is a complex molecular/biological system discovered in 1988 by scientists Allyn Howlett and W.A. Devane [1,2]. The word “Endocannabinoid” was first coined after the discovery of membrane receptors for Δ^9^-tetrahydrocannabinol (Δ^9^-THC or simply “THC”) in 1988 [3]. The ECS plays critical roles in multiple physiological processes such as homeostasis, anxiety, feeding behaviour/appetite, emotional behaviour, depression, nervous functions, neurogenesis, neuroprotection, reward, cognition, learning, memory, pain sensation, fertility, pregnancy, and pre-and post-natal development [4,5,6]. 

In recent years, there has been increasing interest in the role of the ECS in health and disease processes, and its components have been implicated as an emerging target of pharmacotherapy for a wide range of diseases including, but not limited to, general pain, headache, migraine, glaucoma, mood and anxiety disorders, obesity/metabolic syndrome, osteoporosis, neuromotor, neuropsychological and neurodegenerative diseases, respiratory diseases such as asthma, cardiovascular diseases such as stroke, atherosclerosis, myocardial infarction, metabolic disorders, arrhythmias, and hypertension [7,8,9].

Due to the involvement of the ECS in multiple pathophysiological processes, it offers promising opportunities for the development of novel cannabinoids-based therapeutic drugs that may be designed to target different components and/or cell-signalling pathways of the ECS, which may ultimately be of therapeutic benefit. 

Cannabimimetic drugs such as small-molecule cannabinoid receptor agonists and antagonists may be designed to target the ECS and its enzymes and either enhance the bioactivity or activation of endocannabinoids or inhibit their inactivation [3,10]. On the same tangent, blockade of cannabinoid receptor-type 1 (CB_1_R) has been shown to reduce body weight, activation of extracerebral cannabinoid receptors has been shown to alleviate pain, and inhibition of endocannabinoid degradation has been implicated in the modulation of pain and anxiety [11]. 

### 1.2. Components of the ECS

The ECS has increasingly become a favourable target for the treatment of various diseases as many of its components are distributed widely throughout the body and take part in cell-signalling pathways involved in the pathophysiology of many types of diseases.

The components (proteins) of the ECS include receptors, their ligands, and enzymes responsible for their biosynthesis and degradation/deactivation and are widely distributed throughout mammalian tissues and cells [12]. Components of the ECS include: (1) the three main receptor classes that cannabinoids interact with (i) G-Coupled Protein Receptors (GPCRs) (e.g., CB_1_R and Cannabinoid-receptor type 1 (CB_2_R)) and which share 44% overall homology [13], (ii) Ligand-sensitive ion channels (e.g., Transient Receptor Potential Vanilloid 1—TRPV1). TRPV1 is also activated by chemical agents, physical stimuli, capsaicin, and ions, and (iii) Nuclear receptors (e.g., PPARs) [14,15]; (2) the endogenous ligands anandamide or N-arachidonoyl ethanolamine (AEA) and 2-arachidonoylglycerol (2-AG); and (3) the endocannabinoid metabolic enzymes responsible for endocannabinoid synthesis and degradation such as diacylglycerol lipase isozymes α and β, fatty acid amide hydrolase, monoacylglycerol lipase, and *N*-acylphosphatidylethanolamine-selective phospholipase D [3,16]. Refer to Table 1 for components of the ECS. 

## 2. The ECS as a Therapeutic Target

In recent years, genetic and pharmacological manipulation of the ECS has gained significant interest in medicine, research, and drug discovery and development. It’s important physiological and pathophysiological roles offer promising opportunities for the development of novel cannabinergic, cannabimimetic, and cannabinoid-based therapeutic drugs that, genetically or pharmacologically, modulate the ECS via inhibition of metabolic pathways and/or agonism or antagonism of the receptors of the ECS. This modulation results in the differential expression/activity of the components of the ECS—beneficial in a number of diseases. 

### 2.1. Mood and Anxiety Disorders

Anxiety is the body’s natural survival response to harm or dangerous situations, and is characterized by increased responsiveness, defensiveness, and vigilance. Neuropsychiatric/anxiety-related disorders include Panic Disorder (PD), Social Anxiety Disorder (SAD), Generalized Anxiety Disorder (GAD), Post Traumatic Stress Disorder (PTSD), and Obsessive-Compulsive Disorder (OCD) [31]). Globally, these anxiety-related disorders are the most prevalent of any mental disorder. As a result, they are of great social and economic burden. Currently available anxiolytic and anti-depressant agents have limited response rates, limited tolerability, and unfavourable side-effect profiles, thus, cannabinoids may be promising novel alternative therapeutic agents to traditional anxiolytics and anti-depressants. 

Activation of the cannabinoid 1 receptor (CB_1_R) mediates natural rewards (such as social interaction, sexual intercourse, and delicious food) and drug rewards (desirable effects) [32]. As such, the CB_1_R may be a promising, novel drug target for the treatment of mood and anxiety disorders. It is via this receptor that Δ^9^-THC produces the desirable effects on an individual’s mental health, however fleeting. The ECS also potentially modulates synaptic transmission of neurotransmitters, such as mesocorticolimbic dopamine, acetycholine, glutamate, opiate peptides, and GABA, which play significant roles in the control of our emotions and behaviours [33]. The CB_1_R is densely populated in the brain, in areas responsible for the mediation of reward, such as the amygdala, hippocampus, and orbitofrontal cortex [34,35] and, thus, the ECS also plays a role in “emotional metastasis” [32,33]. On the same tangent, single nucleotide polymorphisms (a type of mutation) in the cannabinoid receptor 1 (CNR1) gene that that encodes the CB_1_R has been linked to depression [36,37], nicotine dependence [38], alcohol dependence [39], and possibly other substance-use disorders that are the result of mood and anxiety disorders. 

Cannabidiol (CBD) was first observed to be anxiolytic when it was shown to reverse Δ^9^-THC’s psychotic and anxiogenic effects, via a CB_1_R-independent mechanism [40]. There is strong preclinical evidence that supports CBD’s great potential as an anxiolytic, panicolytic, and anti-compulsive agent. Pre-clinical and animal studies have shown that CBD’s activity decreased condition fear, mitigated the adverse effects of chronic stress, decreases autonomic arousal, prevents fear reconsolidation, and promotes fear extinction [31]. CBD is postulated to regulate fear and anxiety through interaction with the serotonin 5-HT_1A_, the TRPV-1 receptor, and, to a lesser extent, CB_1_R [31]. CB_1_R activation results in anxiolytic effects and plays a role in regulating/preventing fear and preventing chronic stress. CB_1_R seems to mediate the anti-compulsive activity of CBD [31]. Activation of the serotonin 5-HT_1A_ receptor (5-HT_1A_R) by CBD has been implicated in the regulation of fear and prevention chronic stress [31]. Another proposed mechanism of action by which CBD may produce anxiolytic effects is by upregulating hippocampal AEA, an endogenous cannabinoid with anxiolytic properties [41].

A 2011 preliminary study by Bergamaschi and colleagues investigated the effect of a single dose of CBD on subjects undertaking a simulation public speaking test (SPST). A total of 24 patients with Social Anxiety Disorder (SAD), who were never treated prior, received a single 600 mg dose of CBD before the SPS test. There was an improvement in speech performance, a reduction in anxiety, cognitive impairment, and alert anticipatory speech [42].

In murine models, CBD was able to reduce the depression induced by the Forced Swimming Test (FST), tests of conditioned fear, conflict tests, and restraint stress tests [31]. The mechanism of action is suggested to be by activation of the 5-HT_1A_ receptor. It has also been postulated that CBD increases brain-derived neurotropic factor (BDNF), thereby reducing depression [43]. The BDNF protein is responsible for neurogenesis (formation of nerve cells), and the growth, maintenance, and survival of nerve cells. 

### 2.2. Pain Management

Pain is a symptom of many diseases. Both anecdotal and scientific evidence support the use of *C. sativa* L. and its secondary metabolite for overall pain management, and is effective even against chronic pain—both as a stand-alone drug and as an adjuvant, and there is record of the use of *C. sativa* L. in pain management in Chinese pharmacopoeia—some 5000 years ago. 

More recently, the ECS has been implicated in the management of pain as cannabinoids have been shown to target components of the ECS [44] such as the CB_1_R, CB_2_R, non-CB_1_R/CB_2_R cannabinoid G protein-coupled receptor (GPCR) 55 (GPR55) [45], GPCR 18 (GPR18) aka *N*-arachidonoyl glycine (NAGly) receptor [46], opioid/serotonin (5-HT) receptors [47,48,49], TRPV1 [50,51], and PPARα and γ [15]. Additionally, it is notable that, in a murine model, the GPR55 receptor modulates the proinflammatory cytokines IL-4, IL-10, IFN gamma, and GM-CSF, thereby mitigating hyperalgesia [45].

Antagonists of CB_2_R have been reported to demonstrate antinociceptive properties in models of inflammatory and nociceptive pain [52]. One mechanism of action is possibly by inhibition of AEA metabolism; another possibility is via modulation of peroxisome proliferator-activated receptor α agonists, TRPV1 antagonists, and/or α_2_-adrenoceptor modulators [52]. In some cases, this is accomplished via activation of opioid system/enhancement of μ-opioid receptor agonists [52]. On the same tangent, cannabinoid and opioids, and cannabinoids and non-steroidal anti-inflammatory drugs (NSAIDs), have been shown to act synergistically [52]. Current evidence suggests that CBD, in particular, may have therapeutic benefits in treating Rheumatoid arthritis, Fibromyalgia, arthritis, chronic back pain, chronic abdominal pain due to surgery, and chronic pancreatitis, headache, and facial pain. 

Studies in murine models of arthritic pain have also shown great promise [53]. In one animal model, cannabinoids were shown to inhibit neuropathic nociception caused by traumatic nerve injury, disease, and toxic insults [54]. In yet another animal model, cannabinoids demonstrated therapeutic efficacy against thermal pain, noxious pain, post-operative pain, cancer pain, and spinal cord injury-related pain [55]. On the same tangent, the endocannabinoid AEA demonstrated antinociceptive properties at the spinal level [50]. 

In general, *C. sativa* L., and its secondary metabolites thereof, may be a safer, non-addictive alternative to opioids, non-steroidal anti-inflammatory drugs (NSAIDs), and most painkillers. This has contributed to CBD’s growing popularity, particularly in professional sports and cancer-management. Furthermore, CBD is well-tolerated across wide dose ranges. 

CBD could be particularly useful in cases where chronic cancer pain is refractory to treatment with traditional analgesics. A 2018 review article/meta-analysis by Vučković and colleagues explored scientific studies conducted between 1975 and March 2018 to examine CBD’s therapeutic applicability in treating cancer-associated pain, fibromyalgia, and neuropathic pain, and concluded that the current scientific evidence supports the use medical cannabis in pain management [44]. There are many components to the many different types of pain. Vučković and colleagues, 2018, also postulate a number of possible mechanisms of action of CBD-induced analgesia [44]. These include the reduction in inflammation, activation of some pain inhibition pathways, inhibition of neuropeptide and neurotransmitter release, and/or regulation of neuron excitability (particularly in the case of neuropathic pain). 

In the present day, Nabiximols (Sativex^®^), a synthetic cannabinoid oromucosal spray, has been approved in some European countries and in Canada for the treatment of cancer-related pain. It is also used for spasticity and neuropathic pain in patients with Multiple Sclerosis. 

Components of the ECS are also expressed in migraine-related structures [56] and, as such, the ECS may also be a target for the treatment of migraines. Refer to Table 2 for a list of synthetic cannabinoids and their therapeutic window for pain.

### 2.3. Cannabinoids as an Alternative to Opioids 

Opioid overdose (OOD) is a worldwide crisis, primarily due to over-prescription of opioids for the management of chronic pain, and also to the illicit drug market. Opioid overdose accounts for approximately 69,000 deaths worldwide, whereas some 15 million people are addicted [59].

An opioid (narcotic) is a class of drugs manufactured synthetically or from the opium plant. The mechanism of action is by binding to opioid receptors (G protein-coupled) located primarily in the central and peripheral nervous system and the gastrointestinal system. Ligands, the endogenous opioids that bind to said receptors, include endorphins, endomorphins, enkephalins, and dynorphins. These receptors mediate analgesia and nociception, and are typically used as pain relievers and anaesthetics. Other uses are to suppress diarrhoea and coughing, and to relieve shortness of breath. This class of drugs include heroin and synthetic opioids such as Fentanyl (Actiq^®^, Duragesic^®^, Fentora^®^, Abstral^®^, and Onsolis^®^), codeine, Hydrocodone (Hysingla^®^ and Zohydro ER^®^), Hydrocodone/acetaminophen (Lorcet^®^, Lortab^®^, Norco^®^, and Vicodin^®^), Hydromorphone (Dilaudid^®^ and Exalgo^®^), Meperidine (Demerol^®^), Methadone (Dolophine^®^ and Methadose^®^), Morphine (Kadian^®^, MS Contin^®^, and Morphabond^®^), Oxycodone (OxyContin^®^, and Oxaydo^®^), Oxycodone and Acetaminophen (Percocet^®^ and Roxicet^®^), and Oxycodone and naloxone. Fentanyl is 50 to 100 times more potent than morphine [60]. Side effects of opioid abuse include nausea, respiratory depression, sedation, euphoria, constipation, urinary retention, and itchiness. Side effects of opioid overdose include pinpoint pupils, drowsiness, cyanosis, slow breathing, loss of consciousness, and even death. 

The analgesic effects of *C. sativa* L. and its secondary metabolites have made them promising tools in combatting the opioid crisis. This if further confirmed by the presence of cannabinoid receptors in peripheral, spinal, and supraspinal neurons associated with modulation of nociceptive signalling [61,62,63,64,65] and the implication of ECS in opiate dependence withdrawal [48]. In a sample of 4,840,562 persons, the legalization of medical cannabis directly correlated with lower chances of opioid use [66]. 

A preliminary cohort study reported a clinically and statistically significant relationship between enrolment in a New Mexico Medical Cannabis Program (MCP) and pain reduction, opioid prescription cessation (no prescription of opioid medication within the last 3 months), reduction in daily intravenous (IV) injection of opioid medications, reduced hospitalization due to prescription opioid medications (POMs) [67], reduced health care costs [67], and improvements of overall quality of life, social life, concentration, and activity levels [68]. A 41% opioid dose reduction (ODR) was also achieved using medical cannabis in cancer and rheumatological patients [69]. 

An association was also found between a reduction in opioid related deaths in Colorado and the legalization of recreational cannabis in Colorado (increasing access to medical cannabis via dispensaries) [70,71,72,73]. Another found a direct relationship between the implementation of medical cannabis access laws and the reduction in the probability a provider prescribes any opioids net of any offsetting effects, the total number of patients receiving opioids and total days’ supply of opioids prescribed [74]. Other studies suggest that the implementation of more flexible medical and adult-use marijuana laws may directly correlate with a reduction in opioid overdose death rates [75,76] and lower opioid prescribing rates (5.88% and 6.38% lower, respectively) [77].

A 2020 study by Blake explored the prescription rates of opioids in 19 states where medical cannabis is legal [78]. Results of this study show that, in these states, opioid prescriptions decreased. In another study, the decreased opioid use (in persons aged 18–55—Medicare/Medicade populations) was only associated with the implementation of a medical cannabis law (as opposed to a recreational cannabis law) [73,79]. On the same tangent, a 2019 study by Flexon and colleagues report no relationship between medicinal cannabis legislation and opioid misuse [80]. In another study, medical cannabis access and use directly correlated with and increased rate of cessation of injection of opioids [81]. Cannabis may also have a safer side-effect profile, lower abuse potential, and may even be used to treat some side effects of opioid use such as nausea [82]. 

At this point, it is suggested that cannabinoid-based analgesics may be used as an adjuvant, rather than an alternative form of therapy, and may even produce a synergistic result when used in combination with opioid analgesics [83,84,85]. A 2019 study by Capano and colleagues evaluated the effects of CBD hemp extract on opioid use and quality of life in a prospective cohort study in patients suffering from chronic pain. Patients given a CBD-rich extract were able to significantly improve their quality of life, and significantly reduce, or completely cease, the use of opioids [86]. No positive correlation between frequent cannabis use and frequent opioid use (whether illicit or prescribed) for pain was reported in this study. 

On a different tangent, in contrast to opioids, the primary analgesic used to treat cancer-induced bone pain (CIBP) caused by malignant cancers such as breast cancer that tend to invade bone, peripherally restricted CB_1_R agonists such as 4-{2-[-(1E)-1[(4-propylnaphthalen-1-yl)methylidene]-1H-inden-3-yl]ethyl}morpholine (PrNMI), have demonstrated significant alleviation of CIBP [87].

### 2.4. Inflammation

Inflammation may accompany many diseases, including many types of cancers, asthma, and autoimmune disorders such as rheumatoid arthritis, hepatitis, colitis, multiple sclerosis, and common dermatologic conditions. Cannabinoids, in general, are very potent anti-inflammatory agents. Endocannabinoids, such as AEA and 2-AG, and phytocannabinoids, such as Δ^9^-THC and CBD, have demonstrated anti-inflammatory and immune-suppressive properties via CB_1_R and CB_2_R [88]. Cannabinoids have demonstrated the ability to downregulate cytokine and chemokine production and, in doing so, are able to suppress inflammatory responses [88]. As such, both endocannabinoids and phytocannabinoids may be promising tools in the treatment of inflammatory disorders. 

It has been postulated that CBD binds to an adenosine A2A receptor, and decreases inflammation by way of inhibition of adenosine uptake. This has been confirmed in murine models. In another murine model, CBD was able to mitigate LPS-induced inflammation through said A2A receptors. CBD also had the same effect on inflammation in animal models for multiple sclerosis. In yet another murine model, CBD, by way of the TRPV-1 receptor, was able to reduce the levels of pro-inflammatory cytokines (eotaxin1, IL-2, IL-6, IL-12, IL-17, TNF-α, IFC-c, and MCP-1) [89]. AEA is also implicated in the treatment of inflammation [90]. 

It has also been postulated that CBD is a functional antagonist to the GPR55 receptor [91]. Via inhibition of GPR55 receptor activity, CBD may mediate levels of inflammation by controlling the release of pro-inflammatory cytokines IL-12 and TNF-α [92]. Additionally, by binding to and blocking the GPR55 receptor, CBD may exhibit analgesic effects in neuropathic pain, and anti-inflammatory activity in Inflammatory Bowel Disease [92]. 

CBD interacts with the PPAR-y receptor to mitigate beta-amyloid (Aβ)-induced neuroinflammation [92]. Through said receptor, CBD also promotes neurogenesis in the hippocampus. The anti-inflammatory actions of CBD were also reported in murine models of Type 1 Diabetic Cardiomyopathy, Pneumococcal meningitis, Colitis, Alzheimer’s, and Inflammatory Bowel Syndrome [92]. In murine models, CBD also has the ability to decrease Reactive Oxygen Species (ROS), thereby inhibiting inflammation [92]. The extent to which these results in murine models may be applied to humans requires further study.

### 2.5. Cardiovascular Disorders

Studies have shown that cannabinoids, including CBD, have a cardioprotective role—preventing heart damage, reducing the risks thereof, and maintaining a “healthy” heart and vasculature [93]. Cannabinoids have also shown promise against arrhythmias, atherosclerosis, and stroke [94,95]. Studies also show that cannabinoids may lower the risk of cardiovascular diseases, heart attack (myocardial infarction), and injury as a result of reduced/restricted blood flow (ischaemia) [93]. CBD and other cannabinoids have also been shown to cause relaxation of the blood vessel walls (vasorelaxation) [93]. It is suggested that CBD decreases blood pressure, attenuates atherosclerosis, and increases the available nitric oxide by way of PPARy antagonism [93]. Nitric oxide is a neurotransmitter and blood vessel relaxant, that improves blood circulation, reduces blood pressure, regulates heart rate, prevents clogged arteries, regulates contractility of the heart and vascular tone, prevents adhesion of cells to the endothelium, and prevents the formation of blood clots by inhibiting platelet activation. As an anxiolytic agent, CBD mitigates the cardiovascular response when we become anxious or stressed. 

Proposed mechanisms of action by which CBD exerts its activity on the cardiovascular system are by TRPV channel activation, nuclear factor-kB (NFκB), and map kinase (MAPK) pathways [93]. AEA also activates TRPV1, and is implicated in the treatment of cardiovascular disorders [90]. Other cannabinoids may act by way of CB_1_R activation. CBD is also shown to prevent hypotension by inducing arteriolar and venular vasodilation [93]. 

#### 2.5.1. Diabetes 

Diabetes is a metabolic disease characterized by high blood-sugar levels and is a significant risk factor for cardiovascular diseases (CVD) such as stroke, blood vessel disease, and coronary artery disease, as it damages the nerves and the blood vessels of the heart/cardiovascular system and possibly other organs, such as the eyes and kidney [96,97]. The hormone responsible for the regulation of blood glucose is insulin. In Type 1 diabetes, an autoimmune disease, the pancreatic cells that make insulin are attacked and destroyed by the individual’s own immune system. In Type 2 diabetes, the individual becomes resistant to insulin and, as a result, there is an accumulation of sugar in the blood [98].

Both CBD and Δ^9^-tetrahydrocannabivarin (Δ^9^-THCV) a non-psychoactive cannabinoid, have been shown to play a role in lipid and glucose metabolism in animal models, and may be opportunities for glycaemic control in the case of patients with type 2 diabetes mellitus (T2DM) [99]. The CB_1_R has also been implicated as a therapeutic target for the treatment of T2DM, as the ECS has demonstrated a role in insulin resistance characteristic of T2DM [100]. Δ^9^-THCV has been implicated in the clinical management of type 2 diabetes as it has demonstrated the ability to decrease appetite, up-regulate energy metabolism, and increase satiety [101].

CBD also seems to have therapeutic activity against endothelial dysfunction [93]. The endothelium is a layer of single-celled tissue which lines organs, in this case the heart. Endothelial dysfunction is characterized by inflammation, blood clotting (thrombosis), and impaired vasodilation. High glucose intake, as in cases of diabetes, is a cause of endothelial dysfunction. Another proposed mechanism of action of CBD on diabetes is through the upkeeping of the blood–retinal barrier. Disruption of the blood–retinal barrier is characteristic of diabetes [93]. 

#### 2.5.2. Stroke 

The wide distribution of the components of the ECS makes it a promising target in the treatment of CNS diseases/neurological disorders such as strokes [7]. A stroke is a type of cardiovascular disease that is characterized by brain damage and other possible signs and symptoms such as severe headache, loss of coordination, dizziness, confusion, blurred vision and even temporary blindness, slurred speech, and numbness/paralysis of face or limbs [102]. Strokes are the result of a lack of oxygen and nutrients to the brain due to interruption or restriction of blood supply to brain [102]. Types of strokes include: (1) ischemic stroke due to a blocked artery, and (2) haemorrhagic stroke due to a leaking or burst blood vessel [102]. 

Δ^9^-THC has demonstrated positive effects on brain oxygenation and increased hemodynamic blood flow to the prefrontal cortex, and may possibly be beneficial in the treatment of (frontal lobe) strokes [103]. The anti-spastic properties of CBD may also be beneficial for patients with post-stroke spasticity [103].

In in vivo and in vitro animal models, CBD plays a neuroprotective role in the pathophysiology of ischaemic stroke–the most common type of stroke—characterized by blockage of blood vessels in the brain by blood clots. Studies show that CBD increases cerebral blood flow (CBF), thereby reducing the risk of ischaemic strokes [93]. HU-211 has also demonstrated therapeutic promise against CNS diseases [104].

Another proposed mechanism of action of CBD on CBF is through antagonism of the serotonin (5HT_3_) receptor (5-HT_1A_R) [93]. CBD facilitates 5-HT_1A_R signalling in animal models. Yet, another proposed mechanism of action of CBD on strokes is through the upkeeping of the blood–brain barrier [93]. Disruption of the blood–brain barrier is one proposed cause of ischaemic stroke. 

An increased infarct size is characteristic of heart attacks (myocardial infarction). Studies have shown that CBD reduces infarct size by reducing inflammation [93,105,106]. There is also evidence that CBD influences blood cell function, including promoting the survival and migration of white blood cells, mediating programmed cell deaths, and regulating platelet aggregation [93].

### 2.6. Cancer

Cannabinoids have demonstrated well established analgesic, antinauseant, antidepressant, antiemetic, anti-nociceptive, and orexigenic properties and, as a result, they have been studied and utilized in the treatment of cancer patients receiving chemotherapy or radiotherapy, and in AIDS/HIV patients [107,108,109,110]. In addition to the well-established palliative properties that Δ^9^-THC and CBD exert on cancer-related symptomology, several phyto-, endo-, and synthetic cannabinoids all exert their anti-cancer properties via several different proposed mechanisms of action including, but not limited to: induction of apoptosis, autophagy and cell-cycle arrest, inhibition of cancer cell migration, metastasis, angiogenesis, neovascularization, adhesion, and/or invasion [111,112,113,114,115,116,117]. These properties are likely attributed to their role in endocannabinoid signalling pathways involved in cancer processes such as the MEK-extracellular signal-regulated kinase signalling cascade, and the adenylyl cyclase, cyclic AMP-protein kinase-A pathway [113,118]. Ultimately, the use of cannabinoids to target the ECS-signalling involved in the pathogenesis of these cancers, is a very promising target that is currently being given increasing attention in the medical landscape. 

Multiple studies also confirm the direct correlation between the upregulation of said cannabinoid receptors, endocannabinoid metabolic enzymes, and endogenous ligands in cancerous tissue [119,120,121,122,123,124,125].]. Signalling between cancer cells is also shown to be mediated by cannabinoids [119]. One study suggests that the ECS may play a role in tumour suppression [126]. Multiple studies have also demonstrated the apoptotic, anti-metastatic, anti-angiogenic, anti-inflammatory properties of cannabinoid and non-cannabinoid secondary metabolites of *C. sativa* L. This suggests that cannabinoid-based therapeutics may be promising in the treatment of many different types of cancers, in addition to the aforementioned diseases. 

Cannabinoids such as AEA, Met-F-AEA, 2-AG, Δ^9^-THC, CBD, CBDA, HU120, WIN-552122, JWH-133, AME121, and R-(+)-MET have all demonstrated anti-cancer properties in various cancer models such as breast-, lung-, prostate-, testicular-, gastric-, skin-, colon-, bone cancers, and glioblastomas, lymphomas, leukaemias, and neuroblastomas. Mechanisms of action of these cannabinoids in these cancers range from induction of apoptosis and cell cycle arrest, inhibition of DNA synthesis, inhibition of various signalling pathways such as the PI3K/AKT/mTOR/AMPK or the EGF/EGFR, inhibition of angiogenesis, inhibition of tumour growth, tumour regression, and inhibition of metastasis.

## 3. Neurological/Neurodegenerative Diseases

Neurodegenerative diseases are characterized by inflammation and dysregulation of the function of neurons, and in some cases death, resulting from an ongoing/progressive degeneration of neurons [127]. This category of diseases includes amyotrophic lateral sclerosis (ALS), Alzheimer’s disease, Parkinson’s disease, Huntington’s, Batten disease, fatal familial insomnia, and, by some hypotheses, schizophrenia. These diseases are incurable, but cannabinoids have been shown to provide relief to some symptoms associated with said diseases. Cannabinoids are known to play a role in the modulation of inflammation (neuroinflammation), along with providing and enhancing neuroprotection [95,127,128]. In addition, cannabinoids such as CBD have shown analgesic, anxiolytic, and immunosuppressive properties that may help to combat certain neurological disorders [129].

Cannabinoids have been implicated in the modulation of adult neurogenesis in the hippocampus and the lateral ventricles [130,131]. Chronic treatment of the synthetic cannabinoid HU-210 has been shown to enhance the survival and proliferation of cells in murine models of hippocampal neurogenesis while exerting anxiolytic and anti-depressant properties [132]. Other synthetic cannabinoids, such as JWH-133, AM1241, JWH-056, AM251, WIN55,212-2, and URB597, have also demonstrated pro-neurogenic properties [130]. Neurogenesis is the process by which neural stem cells (NSCs) produce neurons (nerve cells). Neurogenesis in the hippocampus influences our capacity to learn and retain memory. Neuroplasticity is the brains capacity for synaptogenesis, which is the structural change/re-wiring of said connections between neurons. Studies show that schizophrenia and other psychiatric disorders physically alter the brain, as characterized by a reduction in the volume of the hippocampus, along with other areas [40]. This is typically as a result of an inhibition of neurogenesis in the hippocampus.

In one study, prolonged CBD administration demonstrated a neuroprotective role against neuroanatomical alterations in the hippocampus, hippocampal volume loss, and even ameliorated brain damage [133]. In murine models, CBD promoted hippocampal neurogenesis, synaptic- and dendritic-remodelling, and prevented autophagy, neurogenic disruption, stress-induced anxiogenesis, THC-induced neurotoxicity, oxidative damage/ROS production, and neuronal damage [40]. 

Cannabinoids may also have potential in the treatment of mood instability associated with neurological disorders, as the ECS has been implicated in pathophysiology of neurological disorders [134]. Although some studies suggest that cannabinoids in general may be promising in the treatment of neurological disorders, others suggest a link between high consumption of recreational cannabis and an increased risk of mental health disorders such as substance dependence—though this is controversial [120,135]. This is, however, likely due to the presence of THC. Further studies are required to clearly elucidate the pro-neurogenic effects of CBD and other cannabinoids in humans. 

Scientific evidence suggests that cannabinoids such as Δ^9^-THC, CBD, WIN55212-2, and CP-55940 may be used to treat various forms of substance abuse such as heroin-, cocaine-, nicotine- and alcohol-abuse and their symptomologies thereof [136].

### 3.1. Schizophrenia 

While some studies suggest that *C. sativa* L. use may increase the risk of developing psychotic disorders and even worsen prognosis and disease burden, likely due to psychoactive compounds [134,137], others suggest non-psychoactive compounds in the plant may have therapeutic efficacy.

The anti-psychotic, anti-inflammatory, and neuroprotective properties of CBD make it a safer, more tolerable, and promising alternative treatment for psychotic disorders such as schizophrenia [134,138,139]. Δ^9^-tetrahydrocannabivarin (Δ^9^-THCV) is another cannabinoid that has gained interest due to its anti-convulsant and non-psychoactive properties [134]. On this same tangent, whole-cannabis extract, or pure Δ^9^-THC, on the other hand, may be less effective due to the psychoactive properties of Δ^9^-THC and possibly other psychoactive cannabinoids present in the whole-cannabis extract mixture, and may even increase the risk of psychosis [140,141]. In some studies, CBD has demonstrated the ability to attenuate Δ^9^-THC-induced psychotic symptoms in healthy patients and symptoms of schizophrenia in schizophrenics [139].

Both SR141716A and CBD have demonstrated antipsychotic properties in dopamine- and glutamate-based models of schizophrenia [142,143,144].

### 3.2. Epilepsy 

Epilepsy is a neurological/central nervous system disorder that is characterized by frequency seizures. Multiple anecdotal and scientific evidence confirm the success of medical cannabis in reducing the frequency of seizure episodes with the use of CBD—this being after the end-of-the-road, i.e., failing therapy with traditional AEDs [145].

In recent years, there has been scientific interest in cannabinoid-based drugs for the treatment of epilepsy, particularly treatment-resistant epilepsy (TRE) and paediatric-onset drug-resistant epilepsy. Phytocannabinoids such as CBD, cannabigerol (CBG), cannabidavarin (CBDV), and Δ^9^-THCV have demonstrated anti-convulsant properties and may be promising opportunities to develop safer alternatives (and even adjuncts) to traditional antiepileptic drugs (AEDs) [146,147,148]. Of these cannabinoids, Δ^9^-THC and CBD have been given the most attention for their anti-convulsant properties [149]. CBD, in particular, is of particular interest as it has it circumvents the psychotropic effects resulting from the activation of CB_1_R [150]. CBD has demonstrated efficacy as an adjunct treatment option in the clinical management of Lennox–Gastaut syndrome (LGS) and Dravet syndrome (DS) as, in multiple studies, it has reduced the frequency of epileptic seizures [149,151,152,153,154,155]. 

Charlotte Figi, a SCNIA-confirmed Dravet syndrome patient, is the most famous cases of medical cannabis being used to treat epilepsy—likely as, at one point, she was the youngest medical marijuana patient, and this caused of a lot of controversy [156]. Charlotte Figi began having seizures at the age of 3 months [156]. By the age of 5 years, she was having up to 300 generalized chronic-tonic seizures (GCTs) seizures per week (50/day), and facing a failing therapy of a cocktail of antiepileptic drugs and a ketogenic diet [156]. She had to be fed through a tube, had motor impairment and cognitive delay and, as a result, had to be assisted with every activity. 

Charlotte began receiving sublingual doses of *C. sativa* L. plant extract—starting with low doses (2 mg CBD/lb per day) and increasing up to 4 mg CBD/lb per day [156]. This extract, made from the Charlotte’s Web strain, had 0.3% Δ^9^-THC, sufficient to avoid psychosis, and high content of CBD. Twenty months later, Charlotte’s seizures were reduced by 90% to 2–3 per month, and she could now walk, talk, and do activities unassisted [156]. Upon the success of her treatment with CBD, Charlotte no longer had to take the antiepileptic drug Clobazam^®^. The preparation also began to improve her autistic behaviour. A reduction in dosages of this preparation resulted in a return of seizures, clearly indicating that the preparation had therapeutic effects.

In 2018, Epidiolex^®^ became the first and currently the only US Food and Drug Administration (FDA)-approved plant-derived CBD-based pharmaceutical preparation developed for the treatment of Lennox–Gastaut syndrome (LGS) and Dravet syndrome (DS).

## 4. Autoimmune Diseases 

Immune system disorders are the result of dysregulation (hypo- or hyper-activity) of the immune system. In particular, autoimmune disorders are characterized by hyperactivity (overactivity) of the immune system, resulting in the production of antibodies that attack the body’s own tissues instead of invading pathogens. Autoimmune disorders include autoimmune encephalitis, chronic inflammatory demyelinating polyneuropathy (CIDP), Guillain–Barré syndrome, Grave’s disease, Hashimoto’s thyroiditis, multiple sclerosis, inflammatory bowel disease (IBD) (e.g., Chron’s disease and ulcerative colitis), systemic lupus erythematosus (SLE), rheumatoid arthritis, myasthenia gravis, vasculitis, type-1 diabetes mellitus, psoriasis, and scleroderma. 

The ECS has been implicated in immunoregulation as endocannabinoids, synthetic cannabinoids (such as Ajulemic acid and JWH-015, SR144528, and WIN55,212-12), and phytocannabinoids (such as Δ^9^-THC and CBD) have demonstrated immunosuppressive properties, primarily by way of apoptosis [88]. The ECS is suggested to have therapeutic implications in a number of autoimmune (and neurological) diseases as components (CB_1_R and CB_2_R) of the ECS have been expressed in microglial cells [157] and distributed throughout the central nervous system (brain and spinal cord) [158]. 

To reiterate, the CB_1_R is densely populated in areas of the brain responsible for learning and memory, coordination, movement, regulation of hormones, sensory perception, reward and emotions, and body temperature [159]. On the other hand, CB_2_R are primarily expressed in the cells of the immune system [159]. This is further confirmed by the immunosuppressive properties of some cannabinoids [160], and the inhibition of production of proinflammatory cytokines [160,161], likely acting through the CB_2_R [162]. Both types of receptors are implicated in the modulation of neurotransmitter and cytokine release [160]. Through interaction with CB_1_R and CB_2_R, cannabinoids demonstrate the ability to induce apoptosis of T cells and macrophages [160]. 

CB_1_R and CB_2_R are expressed in microglial cells at low and high levels, respectively, with the distribution and expression of CB_2_R is suggested to modulate microglial activity [159]. Microglial cells are morphologically, phenotypically, and functionally related to macrophages [159].

In “resting” macrophages, CB_2_R is not detected [159]. Elevated levels of expression of CB_2_R directly correlating to the conversion of microglial cells into a either a “primed” state, where the cells function in chemotaxis, or a “responsive” state, in which these cells carry out antigen processing [159]. In a fully activated state, CB_2_ is expressed at very low levels in macrophages [159]. In addition to primed and activated macrophages, inflammatory macrophages also express the highest levels of CB_2_R [159]. This means that cannabinoids may only have a window during which to carry out their therapeutic function [159]. CB_1_R is only expressed in very low levels in microglia [159].

2-arachidonylglycerol, an endocannabinoid, interacts with CB_2_R to stimulate a chemotactic response, whereas in vivo and in vitro, the exogenous cannabinoids Δ^9^-THC and CP55940 interact with CB_2_R to inhibited microglia from a chemotactic response to *Acanthamoeba culbertsoni,* an opportunistic pathogen responsible for Granulomatous Amoebic Encephalitis [159]. 

The pro-inflammatory properties of cannabinoids have implicated them as possible treatments for inflammation associated with autoimmune diseases such as type 1 diabetes mellitus, multiple sclerosis, and neuropathic pain [160].

### 4.1. Blood–Brain Barrier (BBB) (Also Referred to as the “Blood–Spinal Cord Barrier” (BSCB))

The blood–brain barrier (BBB) is where peripheral blood circulation (and components/chemicals in the blood, thereof), meet the anatomical structures of the brain (central nervous system) [163]. It is essentially a border or defensive barrier between the CNS and circulating blood [163]. Blood vessels play a critical role in delivering oxygen and nutrients to the tissues and organs of the body, maintaining hormone signalling among tissues, removing metabolic waste and carbon dioxide from said tissues, and general neuroprotection [164,165]. Blood vessels of the central nervous system (CNS) make up the blood–brain barrier and regulate CNS homeostasis, the movement of cells, ions, and molecules between the blood and the brain [164]. In maintaining CNS homeostasis, the BBB confers neuroprotection from pathogens and toxic chemicals circulating in the blood. 

Dysregulation of the BBB has been implicated in the pathogenesis of neurological autoimmune diseases such as antiphospholipid syndrome with neurological involvement [166,167,168], chronic inflammatory demyelinating polyneuropathy (CIDP) [169], Guillain–Barré syndrome (GBS) [170,171,172], Alzheimer’s disease [173], multiple sclerosis (MS) [174], and neuromyelitis optica [175,176,177].

The endocannainoid system has been implicated in the modulation of the blood–brain barrier [178], and may likely be a potential target for the treatment and/or clinical management of neurological or psychiatric diseases such as schizophrenia and epilepsy [179]. Both AEA and 2-AG have been shown to regulate (decrease) the permeability in in vivo and in vitro models of ischaemia/reperfusion, chronic head injury, and multiple sclerosis [178,180,181]. In another study, CBD was shown to enhance brain-targeting capacity, that is, the passage of lipid nanocapsules across the BBB in both in vivo and in vitro models of BBB, and thus may be an opportunity for novel CNS drugs [182]. 

CB_1_R and CB_2_R may provide neuroprotection via protection from processes that damage the BBB such as inflammation (CB_2_R-mediated), excitotoxicity (CB_1_R-mediated), and cell death (CB_1_R-mediated) and oxidative stress [165]. In addition to these neuroprotective effects, CB_1_R and CB_2_R have both demonstrated the ability to restore the BBB and even improve BBB integrity, thus further conferring protection against neurological or psychiatric diseases [165]. One mechanism of action by which endocannabinoid receptors confer protection of the BBB is via Aβ-efflux across the BBB [165]. The medical significance of this is that deposition of Aβ, and an inability to clear such depositions, is implicated in Alzheimer’s disease [183]. This is due to a dysregulation of the BBB and the inability of Aβ to be transported across the BBB [183]. This 2013 study by Bachmeier and colleagues also investigated and demonstrated the role of the ECS in transporting Aβ across the BBB, clearing of Aβ across the BBB, reducing deposition of Aβ in the AD brain, and improving cognitive behaviour in animal models of Alzheimer’s disease [183].

### 4.2. Multiple Sclerosis 

Growing scientific and anecdotal evidence suggests that cannabinoids such as Δ^9^-THC demonstrate therapeutic effects against symptoms of multiple sclerosis such as neuropathic pain and spasticity [184,185]. Sativex^®^, an FDA approved synthetic cannabinoid (a combination of Δ^9^-THC and CBD) is an oromucosal spray made from whole-plant cannabis extract that has demonstrated efficacy in the treatment of moderate to severe symptoms of multiple sclerosis (MS) without adverse side-effects, potential of drug tolerance, or potential for abuse or misuse [186,187,188,189]. It is proposed that Sativex improves MS symptomology by significantly reducing spinal excitability and increasing intracortical inhibition [190]. Another study proposes that Sativex is even more effective at improving MS spasticity than first line antispasticity treatment alone [191]. 

In animal models of experimental autoimmune encephalomyelitis (EAE) and multiple sclerosis (MS), cannabinoids were shown to mediate EAE suppression via CB_1_R expressed by neurons [192]. Inflammation associated with EAE was also shown to be controlled by CB_2_R expressed by encephalitogenic T cells [192]. T-cells deficient in CB_2_R exacerbated the clinical course of EAE by increasing the production and proliferation of inflammatory cytokines [192]. In addition, these T-cell were resistant to apoptosis [192]. This CB_2_R activity was confirmed in a study by Stipe and colleagues who investigated the effect of a dinucleotide polymorphism in a human gene on endocannabinoid-induced inhibition of T lymphocyte proliferation [162]. The CB_2_R cDNA 188–189 AA → GG polymorphism is the result of arginine replacing glutamate at amino acid position 63 [162], and the rate of polymorphism is reported to be increased in autoimmune diseases [162]. In conclusion, variation in the gene that encoded CB_2_R is suggested to put an individual at increased risk for autoimmunity [162].

### 4.3. Rheumatoid Arthritis 

Immunomodulatory, immunosuppressive, and analgesic properties make cannabinoids promising therapeutic agents in the management of rheumatoid arthritis [193,194,195]. The CB_2_R is reported to be a target for RA therapy, as suggested by increased expression in synovial tissues from the rheumatoid joints [196]. JWH133, a selective CB_2_R agonist inhibited the production of the inflammatory mediators interleukin (IL)-6, matrix metalloproteinase-3 (MMP-3), and chemokine (C-C motif) ligand 2 (CCL2) by tumour necrosis factor-α-stimulated fibroblast-like synoviocytes (FLS) derived from the rheumatoid joints [196]. JWH133 also inhibited the osteoclastogenesis of peripheral blood monocytes, which also occurs in RA [196,197]. In a murine model of RA, another cannabinoid receptor 2 agonist JWH-015 demonstrated inhibition of pro-inflammatory cytokine interleukin-1β-induced inflammation in rheumatoid arthritis synovial fibroblasts partly via a glucocorticoid receptor [198].

### 4.4. Disturbances of the Bowel and Inflammatory Bowel Disease (IDB)

Inflammatory Bowel Disease (IDB) describes two conditions, Chron’s disease and ulcerative colitis, which are characterized by chronic inflammation of the gastrointestinal (GI) tract. Whereas ulcerative colitis is characterized by ulcers and inflammation and the colon and rectum, Chron’s disease may affect any area of the GI tract from mouth to anus, though most often the small intestines, which become inflamed [199,200,201]. 

In traditional Indian, Chinese, and African medicine, *C. sativa* L. was used regularly for disorders of the GI tract and of the bowel, and is still of interest in the treatment of such diseases. CBD, in particular, has shown therapeutic potential in the management of IDB. 

Components of the ECS are distributed, though differentially, in colonic tissue (epithelium, lamina propria, smooth muscle, and enteric plexi) [202], as revealed by Western blot and immunocytochemistry. CB_1_R are distributed throughout the enteric nervous system (ENS) [203] and the gut-brain axis (GBA), a communication network between the brain and the gut [204,205]. This suggests that disorders of the gastrointestinal (GI) tract may be treated with drugs that target said CB_1_R and cannabinoid signalling in the ENS [203,206]. 

The ECS has been implicated in gastrointestinal physiology and homeostasis, and in the pathogenesis of Inflammatory Bowel Disease as confirmed by anecdotal data, studies in humans, epidemiologic data, murine models of colitis [206,207], and other pathophysiological conditions [208,209,210,211]. Refer to Table 3 for a list of uses and properties of cannabinoids for bowel disorders.

Multiple anecdotal evidence confirms the therapeutic properties of medical cannabis against abdominal cramps, diarrhoea [228,229], and anorexia [219]. Other disturbances and inflammatory disorders of the bowel [219,230,231,232], such emesis, anorexia, diabetic gastroparesis [233], colitis [234], and colon cancer [235].

There is increasing interest in the use of medical cannabis (and its cannabinoids, particularly Δ^9^-THC, CBD, and CBG) as an alternative to opioids in the treatment of IBD, due to its safer side-effect profile and lower chance of dependency and mortality [212]. 

In addition to Chron’s disease, the ECS may also be a promising therapeutic target for the treatment of functional bowel diseases such as irritable bowel syndrome and secretion- and motility-related disorders of the GI tract [209]. The ECS may also play a protective role against colonic inflammation [208]. It is, however, unclear whether the mechanisms of cannabinoids against IBD is through inhibition of an inflammation pathway or via masking of IBD symptoms [207]. In a murine model, the ECS provides GI tract protection from inflammation and excessive enteric and gastric secretions [209]. On the tangent of murine models of colitis, two novel ligands, CB13 and AM841, may be used by the cannabinoid system in the pathogenesis of inflammatory bowel diseases [206]. 

To reiterate, CB_2_R are primarily expressed in the cells of the immune system [159] and may play a role in mucosal immunity [210]. This is further confirmed by the immunosuppressive properties of some cannabinoids [160], and the inhibition of production of proinflammatory cytokines [160,161], likely acting through the CB_2_R [162]. This suggests a possible role of the CB_2_R in regulation of inflammation of the GI tract, including colitis-associated inflammation [202].

The ECS is proposed to play an immunomodulatory role in gastrointestinal inflammatory disorders [210]. The distribution of CB_2_R in the GI tract suggests that it may also play a role in limiting visceral sensitivity and pain and in the regulation of gastrointestinal propulsion [211]. Methanandamide (MAEA), a non-hydrolysable AEA analog is reported to have effects on the mucosal proinflammatory response, by downregulating the pro-inflammatory cytokines interferon-γ and tumour necrosis factor-α [236]. Inflamed IBD mucosa expressed significantly lower levels of the endocannabinoid AEA [236].

In a study by Storr and colleagues, it is reported that drugs that targeted blocked degradation of the ECS, including the expression fatty acid amide hydrolase (FAAH), may be promising candidates for drugs used to treat IBD [24,25].

In a separate study by Storr and colleagues, CB_2_R-deficient murine models of trinitrobenzene sulfonic acid (TNBS)-induced colitis were administered intraperitoneal injections of the CB_2_R agonists JWH133, AM1241, or the CB_2_R antagonist AM630 [234]. After a 3-day treatment, AM630 demonstrated complete exacerbation of colitis, while JWH133 or AM1241 significantly reduced colitis [234]. In a separate study, Storr and colleagues also reported that drugs that targeted blocked degradation of the ECS, including the expression fatty acid amide hydrolase (FAAH), may be promising candidates for drugs used to treat IBD [24,25].

On the other hand, CB_1_R are widely distributed within the GI tract, particularly in sensory terminals of vagal and spinal neurons and neurons of the enteric nervous system [209]. It has been reported that CB_1_R plays a role in the modulation of multiple GI tract functions such as gastric secretion and emptying, and intestinal motility [209]. It should also be noted that an increased expression of CB_1_R directly correlated with Croton oil-induced intestinal inflammation in a murine model of inflammation [235]. Wright and colleagues also report that cannabinoids demonstrated the ability to enhance epithelial wound closure via interaction with the CB_1_R [210]. The CB_2_R, though less present and not yet well characterized, is also found in the GI tract. CB_2_R may also be a promising therapeutic target due to its non-psychoactive nature, and its immunomodulatory function in inflammatory pathways [213].

In a study investigating the effects of the cannabinoid agonists CP 55,940 and cannabinol on intestinal motility, both cannabinoid agonists demonstrated a delay in intestinal motility [235]. Δ^9^-THC, Δ^11^-THC, cannabinol, and nabilone (but not CBD) were also reported to have the same effect on intestinal motility (gastrointestinal propulsion/emptying) [237]. 

Anecdotal evidence and a prospective placebo-controlled study report that medical cannabis has significant therapeutic effects against Chron’s disease [230,238]. Patients with Crohn’s disease who did not respond to treatments with anti–tumour necrosis factor-α agents, immunomodulators or steroids, responded to treatment with 115 mg of Δ^9^-THC, which significantly mitigated the symptoms of Crohn’s disease, despite its inability to induce remission [220].

CBD, which possesses many of the anti-IBD properties as other cannabinoids, may be more favourable as an anti-IBD drug than Δ^9^-THC due to its antipsychotic properties [239].

## 5. Medical Cannabis in Dermatology

The skin is our largest organ, and its primary role is as a first line defence against external agents. All components of the ECS are found in the skin [240], further establishing the role of the ECS in healthy and diseased skin and general homeostasis [241,242]. Dysregulation of these components is implicated in the pathogenesis of several cutaneous disorders [241].

The use of *C. sativa* L. for skin pathologies has its roots in traditional Chinese medicine where the plant preparations were used as topicals to treat hair loss, skin rashes, ulcers, and wounds [227,243,244,245]. Modern clinical studies also report that cannabinoids demonstrate significant therapeutic effects against skin lesions [246], skin burns [247], and pruritus in several dermatologic diseases such as allergic contact dermatitis, atopic dermatitis, asteatotic eczema, and prurigo nodularis [248]. The Japanese also used *C. sativa* L. (*asashijingan*) to treat skin pathologies caused by poisonous bites and intestinal parasites [249,250].

*C. sativa* L. preparations (powdered leaves) were also used in traditional Arab medicine to treat diseases of the skin such as pityriasis and lichen planus [243,251]. *C. sativa* L. plant preparations including hemp seed oil have also been traditionally used to treat varicose eczema, acne rosea, and scabies [252].

Inflammatory skin disorders such as acne vulgaris, allergic contact dermatitis, dermatomyositis, psoriasis, and scleroderma are a great disease burden globally, and may greatly impact an individual’s self-esteem, social interactions with others, and general quality of life, particularly if accompanied by pain, pruritus, and permanent scarring [253]. Cannabinoids may also have therapeutic application against asteatotic dermatitis, atopic dermatitis, cutaneous manifestations of systemic sclerosis hidradenitis suppurativa, Kaposi sarcoma, and skin cancer [254]. In a murine model, peripheral administration of 0.01 ng AEA inhibited the induction of, and attenuated, carrageenan-induced hyperalgesia, inhibited capsaicin-induced plasma extravasation, and inhibited inflammation via inhibition of neurosecretion from capsaicin-sensitive primary afferent fibres, all via interaction with CB_1_R [255]. Cannabinoid receptors are also found in the skin, and play a role in regulating skin growth and maintaining homeostasis of skin cells (melanocytes, keratinocytes, and sebocytes) [256]. Phytocannabinods such as CBD and Cannabigerol (CBG) have been shown to regulate the expression of epidermal differentiation genes (i.e., involucrin, tranglutaminase, and keratins) [257].

The anti-inflammatory properties of some cannabinoids, particularly CBD, suggest that it may have therapeutic application against dermatological inflammatory diseases [258]. Remember that inflammation plays a role in the pathogenesis of many cancers. This, in addition to other anticancer/anti-neoplastic properties of cannabinoids suggest that they may also play a role in regulating, or at least inhibiting skin carcinogenesis [258]. In addition to anti-inflammatory effects, cannabinoids interact with the ECS components of the skin to produce antipruritic, anti-ageing, anti-cancer [259], and antinociceptive effects [260]. Additionally of note is that solar UV radiation is also shown to induce skin inflammation and carcinogenesis via activation of CB_1_R and CB_2_R [261]. This was confirmed in a murine model with CB_1_R and CB_2_R deficiency which demonstrated significant resistance to UVB-induced inflammation and reduction in UVB-induced skin carcinogenesis [261]. CB_1_R activated by cannabinoids may also play a role in maintenance of epidermal integrity and permeability [262].

Cannabinoids have also been implicated in the treatment of cutaneous autoimmune diseases such as scleroderma, psoriasis, eczema, and atopic dermatitis. These are discussed in the following sections.

### 5.1. Acne

Acne is a chronic inflammatory cutaneous disorder and is the most prevalent skin disorder, globally. It is characterized by the clogging of oil glands in the skin by oil and dead skin cells, resulting in the formation of pimples. According to immunologist Dr. Tamas Biro, CBD inhibits lipid synthesis and induces cell death in human sebaceous gland-derived sebocytes and ultimately may be a safer treatment for acne than Accutane, a traditional drug used to treat severe acne [263].

A study by Dobrosi et al. reported that CB_2_R are expressed in human SZ95 sebocytes, and that the endocannabinoids AEA, and 2-arachidonylglycerol induced upregulation of lipid synthesis, leading to acne. Dobrosi and colleagues also found that inhibiting the said CB_2_R decreased lipid production in said skin cell line [264]. Thus, drugs that inhibit eCB uptake will increase endocannabinoid levels, resulting in a homeostatic production of sebaceous lipids and an anti-inflammatory response that may be beneficial in treating cutaneous inflammatory conditions and dry skin [265]. In 2014, Oláh and colleagues explored the effects of CBD on human sebaceous gland function and discovered that CBD exerts sebostatic and anti-inflammatory effects on human sebocytes. That is, CBD was shown to have lipostatic action and even decreased sebocyte proliferation. In this same study, CBD was able to inhibit pro-acne agents, such as arachidonic acid (AA), a combination of linoleic acid and testosterone (LA-T), AEA, 2-arachidonylglycerol, that induced excessive lipid synthesis in human sebocytes, leading to acne [266].

Although current scientific is limited, existing evidence suggests that CBD has a positive safety profile in dermatology. Anecdotal evidence also suggests that CBD may also help with anti-aging/wrinkles. This may be attributed to its antioxidant activity. CBD may also help with the natural healing process for open sores caused by dried and cracked skin.

### 5.2. Psoriasis 

Psoriasis is a chronic hyperproliferative, inflammatory skin disease characterized by up-regulation of the keratins K6 and K16 [267]. Psoriasis is also accompanied by increased keratinocyte proliferation and differentiation [268], that is the result of dysregulation of Th1 and Th17 immune cells in the skin, T-cell infiltration, neutrophil infiltration, and activation of dendritic cells and macrophages [243,269]. This suggests that, as cannabinoids regulate Th1 and Th17 immune cells in the skin, the ECS might be a promising therapeutic target for psoriasis [270].

The endocannabinoid AEA, and the CB_1_R-specific agonist, arachidonoyl-chloro-ethanolamide (ACEA) are also shown to inhibit epidermal differentiation and the proliferation of epidermal keratinocytes (immature skin cells) [267,271] via downregulation of the expression of keratins K6 and K16 in vitro and in vivo [271]. In immortalized human keratinocytes (HaCaT) and normal human epidermal keratinocytes (NHEK), AEA demonstrated inhibition of cornified envelopes, characteristic of keratinocyte differentiation [267]. The anti-inflammatory properties of AEA may also be due to its ability to inhibit cytokines produced by keratinocytes [272].

These immature skin cells are characteristic of psoriasis [267,273]. This mechanism of action is via the activation of the CB_1_R, which inhibits human hair growth and decreases proliferation of epidermal keratinocytes [267].

### 5.3. Eczema 

Eczema is a skin condition characterized by patches of itchy, cracked, rough, and inflamed skin, typically caused by allergens, microbes, extreme temperatures, hormones, stress, dietary intake, or irritants [274]. The anti-inflammatory, anti-pruritic, anti-itching, pro-neoplastic, moisturizing, and anti-oxidant properties of *C. sativa* L., particularly CBD, has made medical cannabis a promising and safe alternative to traditional dermatological drugs [259,275,276,277].

In a study by Maghfour and colleagues, researchers investigated the efficacy of topicsal CBD in the treatment of inflammatory skin disorders such as eczema [278]. Using the Patient Oriented Eczema Measure (POEM) and the Quality-of-Life Hand Eczema Questionnaire (QOLHEQ), subjects self-reported a significant reduction in eczema severity, reduction in the psychosocial burden of eczema, reduction in the emotional burden of eczema, decreased itching, and overall improvement of eczema [278].

### 5.4. Fibrotic Skin Diseases 

Systemic scleroderma (simply “sclerosis”/“sclero” = hard; “derma” = skin) is a rare, chronic, autoimmune rheumatic disease characterized by a connective tissue disorder that causes the skin and connective tissues to harden and tighten, and may also affect surrounding muscles, blood vessels, heart, lungs, kidneys, and the digestive tract [279]. A number of factors may cause sclerosis, including an attack of one’s connective tissues by one’s own immune system (“an autoimmune attack”), drugs and certain medications, microbes, and genetics [279]. The endocannabinoid has been implicated in the pathogenesis of dermal fibrosis (scleroderma) [280] via the cannabinoid receptor CB_2_R. In a CB_2_R-deficient murine model of bleomycin-induced fibrosis, selective agonists and antagonists of CB_2_R were administered and evaluated for their effect on the dermal thickness and number of infiltrating leukocytes in lesional skin [280]. In comparison to wildtype mice with CB_2_R (CB_2_R(+/+)), mice deficient in CB_2_R (CB_2_R(−/−) were more sensitive to bleomycin-induced dermal fibrosis, and demonstrated increased dermal thickness [280]. The CB_2_R antagonist AM-630 increased dermal thickness and leukocyte infiltration in lesional skin, whereas CB_2_R agonist JWH-133 reduced leukocyte infiltration and dermal thickening [280].

Δ^9^-THC is also suggested to have anti-fibrotic events in a murine model via interaction with the CB_1_R, possibly by medication of leukocyte infiltration [281]. This was confirmed with CB_1_R-deficient (CB_1_R(−/−)) mice that demonstrated resistance to/protection from bleomycin-induced dermal fibrosis, with reduced dermal thickening, myofibroblast counts, and hydroxyproline content [281]. On the other hand, ACEA-induced CB_1_R activation resulted in increased fibrotic thickening to bleomycin and increased leukocyte infiltration [281]. It should be noted that in a TSK-1 mouse model, CB_1_R knockout (via FAAH inhibition) did not prevent fibrosis [281], and increased levels of cannabinoids were able to induce fibrosis via CB_1_R [243].

As CBD interacts primarily with the CB_2_R, CBD may be a good candidate for treatment of sclerosis, while as Δ^9^-THC interacts with mesenchymal cells and immune cells via CB_1_R and CB_2_R, Δ^9^-THC may be a good candidate for the treatment of systemic sclerosis [281]. The PPARγ receptor may also be a potential target for treating bleomycin-induced scleroderma [242]. VCE-004.8, a non-thiophilic and chemically stable derivative of the CBD quinol and a dual agonist of PPARγ and CB_2_R, showed promising anti-fibrotic efficacy in a murine model of bleomycin-induced scleroderma, and demonstrated reduction in dermal thickness, reduction in blood vessels collagen accumulation, inhibition of mast cell degranulation, inhibition of macrophage infiltration in the skin, inhibition of TGFβ-induced Col1A2 gene transcription and collagen synthesis, and inhibition of TGFβ–mediated myofibroblast differentiation and wound-healing activity [242]. The expression of many genes linked to fibrosis was also shown to be downregulated by VCE-004.8 [242].

A synthetic cannabinoid, WIN55,212-2, administered 1 mg/kg/day, demonstrated complete prevention of bleomycin-induced scleroderma in a murine model, while also downregulating markers of fibroblast activation such as including α smooth muscle actin and the profibrotic cytokines transforming growth factor (TGF)β, connective tissue growth factor (CTGF) and platelet-derived growth factor (PDGF)-BB [282].

## 6. Eating Disorders

The ECS has also been implicated in normal appetite control, determination of appetitive value, weight regulation and obesity [283], as confirmed by cannabimimetic drugs that interfere with the ECS and thus influence obesity [284]. Anecdotal evidence has long confirmed that *C. sativa* L. will likely cause “munchies” after smoking. On the same tangent, Δ^9^-THC, AEA, and 2-AG have been implicated in appetitive processing [283,285,286,287,288]. It is on this basis that synthetic THC drugs such as Dronabinol^®^ and Nabilone^®^ have been designed to treat chemotherapy-associated nausea and vomiting, and anorexia in cancer (and HIV/AIDS patients) patients. 

In rodent models, Δ^9^-THCV, has been implicated in the clinical management of obesity and it has demonstrated the ability to decrease appetite, up-regulate energy metabolism and increase satiety [101]. SR141716A ((N-(piperidin-1-yl)-5-(4-chlorophenyl)-1-(2,4-dichlorophenyl)-4-me thyl-1H-pyrazole-3-carboxamide hydrochloride)), a potent and selective antagonist of the brain cannabinoid receptor CB_1_R, widely expressed in the brain [289], has demonstrated the ability to influence ingestive behaviours [290], and suppress the food intake of a very highly palatable cane-sugar mixture in marmosets [291]. SR141716A has also demonstrated the ability to modulate, by dose, motivation, and locomotor activity (“work”) to consume alcoholic beverages [292]. This implicates SR141716A as a potential to treat alcoholism [292]. Cannabinoid CB_1_R agonist CP 55,940 ((-)-cis-3-[2-hydroxy-4-(1,1-dimethylheptyl)phenyl]-trans-4-(3-hyd roxypropyl)cyclohexanol) has also been shown to stimulate an appetite for palatable beverages [293].

Implication of the ECS in appetite provides some explanation for the crave (“munchies”) ravenous eating that often accompanies the smoking of the *C. sativa* L. plant. On the same tangent, Δ^9^-THC has been reported to have hyperphagic properties; however, this is inhibited by CBD [294].

### Anorexia Nervosa

Anorexia is a potentially life-threatening psychological and eating disorder characterized by a distorted perception of body type, body shape/proportion, and body weight that often leads to depression, intense fear of weight gain, self-starvation, and extreme weight loss. A significant number of morbidity cases in cancer patients is often caused by anorexia. Eating disorders may be due to an impairment in endocannabinoid signalling [295] as evidenced by an upregulation of CB_1_R mRNA in the blood of patients with anorexia nervosa and bulimia nervosa [295], and significant reduction in body weight loss and running wheel activity in an activity-based anorexia (ABA) rodent model after administration of CB_1_R/CB_2_R agonist Δ^9^-THC [296,297] and the synthetic CB_1_R/CB_2_R agonist, CP-55,940 [296]. 

Δ^9^-THC is a known orexigenic (appetite stimulant), as confirmed by thousands of years of anecdotal evidence and modern-day clinical studies. Increased eating leads to increased rate of weight gain, which ultimately combats cachexia. CBD is also a known orexigenic agent. A 2008 study by Costiniuk and colleagues evaluated and reported the efficacy of oral cannabinoid-containing medications (OCs) for the management of interferon and ribavirin-induced anorexia, nausea, and weight loss in patients with chronic hepatitis C virus [298]. The mechanism of action of antiemetic and antinauseant activity of both Δ^9^-THC and CBD is unclear, but may be due to a direct effect on gastrointestinal function, central antiemetic properties, and/or psychological changes [237].

## 7. HIV/AIDS-Related Disorders

CBD is used to alleviate the wasting syndrome associated with HIV and AIDS [299]. It is used as an antiemetic and orexigenic agent (appetite stimulant) and may generally improve the overall quality of life of an HIV/AIDS patient. Both anecdotal and clinical evidence suggest that CBD in HIV/AIDS patients may improve appetite, reduce nausea and vomiting, increase caloric intake, promote weight gain, improve memory and dexterity, improve mood, and mitigate the negative side effects of current anti-retroviral [299] and therapeutic agents. In terms of disease progression (morbidity) and delaying the likelihood death from HIV/AIDS, current studies show that CBD is not effective [299]. 

Δ^9^-THC may also be used as an antiemetic and orexigenic agent (appetite stimulant) and may generally improve the overall quality of life of an HIV/AIDS patient, and ultimately alleviate the wasting syndrome associated with HIV and AIDS. Dronabinol^®^, a synthetic, Δ^9^-THC product approved by the FDA in 1985, is used to treat anorexia and weight-loss in HIV/AIDS patients. 

## 8. Cannabinoids for the Treatment of Hepatitis B Virus

Liver disease, in general, is a major global health burden. Viral hepatitis is a disease of the liver characterized by liver inflammation and damage as a result of viral infection. Viral hepatitis is commonly caused by one of five hepatotropic viruses (hepatitis A, B, C, D, and E), but may be caused by other viruses such as the herpes simplex virus (HSV), yellow fever virus (YFV), cytomegalovirus (CMV), and Epstein–Barr virus (EBV). Hep A, Hep B, and Hep C are the most common causes of viral hepatitis. Hep A and Hep E are spread by the faecal–oral route, that is, via contaminated food or water. Hep B, Hep C, and Hep D are spread through blood transfusion. There is evidence that these may also be spread sexually. 

Hepatitis may also be caused by other types of micro-organisms, including bacteria, fungi, and even parasites, non-infectious agents such as drugs and alcohol, and other metabolic and autoimmune diseases [300]. Hepatitis infections may either be acute (short-term), where the body will be able to resolve the infection, or chronic (long-term), where the body is unable to resolve the infection, resulting in liver failure, liver cirrhosis, and liver cancer. 

In a 2017 in vitro study by Lowe and colleagues, CBD was shown to have inhibitory effects against Viral Hepatitis C (HBC) but not Viral Hepatitis B (HBV). In a dose–response assay, at a single concentration of 10 µm, CBD was able to dose-dependently inhibit HCV replication by 86.4% [301]. CBD also seems to have therapeutic efficacy against autoimmune/non-viral hepatitis [301]. CBD shows in vivo activity through its interaction with the CB_2_R. This interaction inhibits the pathogenesis of autoimmune hepatitis by inducing the apoptosis of thymocytes and splenocytes. This, in turn, inhibits T-cells and macrophages attacking the liver, thereby inhibiting the release of pro-inflammatory cytokines [301]. 

Myeloid-derived suppressor cells (MDSCs) are responsible for regulating the immune system by suppressing T-cell function and inhibiting liver inflammation. Through interaction TRPV1 receptor, CBD is shown to activate MDSCs, thereby inhibiting inflammation and hepatitis in a murine model [302]. In a concanavalin A model of acute hepatitis in mice, Hegde and colleagues report that CBD was able to reduce ConA-induced inflammation by inhibiting the production and release of various pro-inflammatory cytokines, protecting the mice from acute liver injury [302]. CBD was also shown to mitigate liver fibrosis, a characteristic scarring of healthy liver tissue, that is a result of untreated viral hepatitis. In said study, Lowe and colleagues discovered that CBD inhibited activated hepatic stellate cells (HSCs) that play a role in the development and progression of liver fibrosis. 

## 9. Cannabinoids Used to Modulate the ECS in Cannabinoid-Research 

The role of the ECS in the development of various diseases, multiple ECS targets and multiple types of cannabinergic, cannabimimetic, and cannabinoid-based lead-compounds have been established and studied extensively. Refer to Table 4, Table 5, Table 6, Table 7, Table 8, Table 9, Table 10, Table 11, Table 12 and Table 13 for some types of compounds that may modulate the ECS in the treatment of various disorders and diseases. 

Regarding the use of central CB_1_R agonists [136], examples of CB_1_R agonists are listed in Table 5 below.

**Table 5:** Examples of CB_1_R agonists and their therapeutic windows.

	Central CB_1_R Agonists	Biological Effect(s) and/or Mechanism of Action	Reference
i.	Δ^9^-THC (partial agonist)	- Anticancer - Anti-microbial - Anti-inflammatory - Analgesic	[88,305,306,307,308,309,310,311,312,313,314,315,316,317,318,319,320,321]
ii.	WIN55,212-2 (also a CB_2_R agonist)	- Decreases the severity of seizures in rodents - Prevents anhedonia in rodents - Anti-cancer properties	[322,323,324] [325,326,327,328]
iii.	ACPA (Arachidonylcyclopropylamide)	- Anti-depressive - Anxiolytic - Anti-nociceptive in mice	[329,330,331]

The use of allosteric modulators of CB_1_R. Examples of *CB_1_R* allosteric modulators are listed in Table 6 below.

**Table 6:** Examples of CB_1_R allosteric modulators and their therapeutic windows.

	CB_1_R Allosteric Modulators	Biological Effect(s) and/or Mechanism of Action	Reference
i.	**GAT211** (positive allosteric modulators (PAM)(racemic))	- Anti-psychotic - Anti-nociceptive/analgesic in models of neuropathic and/or inflammatory pain	[332,333,334,335,336]
ii.	**GAT228** (R-enantiomer)	- May improve Huntington’s disease (HD) symptomology - Reduces corneal inflammation and ocular pain.	[336,337,338]
iii.	**GAT229** (S-enantiomer)	- May improve Huntington’s disease (HD) symptomology	[336,337]
iv.	**ORG27569** (negative allosteric modulator (NAM))	- Reduces cocaine and methamphetamine seeking behaviour in rat model - Hypophagic, and thus may have use in the treatment of obesity	[339,340,341,342,343,344]

Peripheral CB_1_R agonists do not cross the blood–brain barrier (BBB), and are suggested to circumvent the psychotropic effects and other adverse side-effects such as cardiovascular and immune perturbations produced by CB_1_R activation. Examples of peripheral CB_1_R agonists (aka peripherally restricted cannabinoid 1 receptor (PRCB)) are listed in Table 7. 

**Table 7:** Examples of peripheral CB_1_R agonists (aka peripherally restricted cannabinoid 1 receptor (PRCB)) and their therapeutic window.

	Peripheral CB_1_R Agonists (Aka Peripherally Restricted Cannabinoid 1 Receptor (PRCB))	Biological Effect(s) and/or Mechanism of Action	Reference
i.	4-{2-[-(1E)-1[(4-propylnaphthalen-1-yl)methylidene]-1H-inden-3-yl]ethyl}morpholine (“PrNMI” aka 2-“5u”	- Anti-allodynic properties (suppresses CIPN* mechanical and cold allodynia in a dose-dependent way). *Chemotherapy-induced peripheral neuropathy (CIPN) - Alleviation of cancer-induced bone pain (CIBP) - Neuropathic pain	[87,345,346]
ii.	4-{2-[(1E)-1-[(4-Methoxynaphthalen-1-yl)methylidene]-1H-inden-3-yl]ethyl}morpholine (2-5j)	- Anti-allodynic properties (suppresses mechanical allodynia symptoms	[346]
iii.	2-5j (2-5j)	- Anti-allodynic properties (suppresses mechanical allodynia symptoms	[346]

Peripheral CB_2_R agonist is used to circumvent the psychotropic effects and other adverse side-effects such as cardiovascular and immune perturbations produced by CB_1_R activation [136]. Examples of peripheral CB_2_R agonists are listed in Table 8 below.

**Table 8:** Examples of peripheral CB_2_R agonists and their therapeutic windows.

	CB_2_R Agonists	Biological Effect(s) and/or Mechanism of Action	Reference
i.	AM1241 (University of Connecticut)	- Analgesic - Anti-inflammatory - Reduction in bone resorption (loss) in NCTC-2472 bone sarcoma cell line - Attenuation of spontaneous and evoked pain in tumour-bearing limb - Reduction in cancer-induced pain - Neuropathic pain	[136,347,348,349,350,351]
ii.	A-76260	- Analgesic in murine model	[352]
iii.	HU-308 (Hebrew University)	- Neuropathic pain - Anti-inflammatory - Analgesic - Osteoprotective - Prohomeostatic	[353,354,355]
iv.	GSK554418A	Acute/chronic pain	[356]
v.	GW842166X	Inflammatory pain	[357]
vi.	GW405833	- Anti-inflammatory - Suppresses neuropathic pain	[358]
vii.	GP1a	- Anti-depressant - Decreased severity in experimental cystitis - Antiallodynic effects in animals on retrovirus infection-induced neuropathic pain - Modulation of HIV-1-associated neurocognitive disorders (HAND)	[359,360,361]
viii.	JWH015	- Antiallodynic effects in animals on retrovirus infection-induced neuropathic pain - Attenuates bone cancer pain - Anti-inflammatory - Immunosuppressive - Anti-obesity	[198,360,361,362,363,364,365]
ix.	JWH133	- Antiallodynic effects in animals on retrovirus infection-induced neuropathic pain - Alleviates fibrosis in murine model - Anti-inflammatory - Anti-proliferative and anti-angiogenic in non-small lung cancer cells (A549) and human umbilical vein endothelial cells. - Cardioprotective against ischemia/reperfusion-induced apoptosis - Reduces neurodegeneration, neuroinflammation, and spatial memory impairment in Alzheimer’s disease model - Anti-nociceptive	[360] [366,367,368,369,370]

Regarding the use of CB_1_R antagonists [136], of note is that side effects of CB_1_R antagonism may include neuropsychiatric sequalae (e.g., anhedonia and anxiety), pain, hyperalgesia, hypertension, and pro-convulsive effects [136,371]. Examples of CB_1_R antagonists are listed in Table 9 below.

**Table 9:** Examples of peripheral CB_1_R antagonists and their therapeutic windows.

	CB_1_R Antagonists	Biological Effect(s) and/or Mechanism of Action	Reference
i.	SR141716A (Rimonabant)—the first developed CB_1_R antagonist. Now discontinued due to unwanted side effects such as depression, anxiety, and suicidal thoughts.	- Obesity possibly via inducing loss of appetite or increase in metabolic rate (loss of fat mass) via interaction with corticotropin-releasing hormone (CRH), a known anorexigenic - Rimonabant inhibits CB_1_R activation which is responsible for lipogenesis - Tobacco addiction - Inhibition of cannabinoid-induced heroin-seeking behaviour in rats	[6,136,372,373]
ii.	AM251	- Attenuates mechanical allodynia - Attenuates thermal hyperalgesia - Anti-nociceptive - Anti-depressive effects - Improves recognition memory in murine model - Anti-cancer/modulation of tumour growth in mice	[374,375,376]
iii.	SLV-326 (Solvay)	- May have anti-obesity, anti-addiction, anti-depressant, and anxiolytic effects	[136]
iv.	LY320135 (Lilly)	- May have anti-obesity, anti-addiction, anti-depressant, and anxiolytic effects	[136,372,377]
	**Neutral Antagonists**		
v.	AM4113	- Prevents opioid addiction (self-administration) in rodent model - Anti-depressant - Anxiolytic - Prevents relapse to nicotine-seeking behaviour in rats - Anti-obesity via suppression of appetite - Regulate body weight in rats - Anti-nauseant	[136,378,379,380,381,382]
vi.	O-2654 (Organix)	- May have anti-obesity, anti-addiction, anti-depressant, and anxiolytic effects	[136]
vii.	AM5171 (University of Connecticut)	- May have anti-obesity, anti-addiction, anti-depressant, and anxiolytic effects	[6,136,272,338,373]

Examples of endocannabinoid-like compounds (fatty-acid ethanolamides) that interact with receptors outside of CB_1_R and CB_2_R [136,383] are listed in Table 10.

**Table 10:** Endocannabinoid-like compounds (fatty-acid ethanolamides) that interact with receptors outside of CB_1_R and CB_2_R.

	Endocannabinoid-Like Compounds (Fatty-Acid Ethanolamides)	Biological Effect(s) and/or Mechanism of Action	Reference
i.	OEA (an endogenous PPAR-α agonist)	- Satiety-induction - Weight reduction - Anti-inflammation Via binding to peroxisome proliferators-activate receptor-α (PPAR- α)	[136]
ii.	Palmitoylethanolamide (PEA)	- Anti-inflammation	[136]
iii.	*N*-oleoyl-ethanolamide	May act as an alternative substrate for FAAH, and in doing so, inhibit the degradation of AEA	[383,384]
iv.	*N*-linoleoyl-ethanolamide	May act as an alternative substrate for FAAH, and in doing so, inhibit the degradation of AEA	[383,384]
v.	*N-*arachidonoyl-glycine	May act as an alternative substrate for FAAH, and in doing so, inhibit the degradation of AEA	[384,385,386]
vi.	*N*-acyl-taurine	May act as an alternative substrate for FAAH, and in doing so, inhibit the degradation of AEA	[383,384,387]
vii.	*N*-palmitoyl-ethanolamide	Reduced expression of FAAH	[384,388]

Synthetic cannabinergic agonists include CP-55940 (Pfizer), HU-210 (Hebrew University), WIN55212-2 (Winthrop), a cannabinoid agonist by Novartis for neuropathic and inflammatory pain treatment, BAY-387271 (Bayer) for stroke, and AM356 [136]. Refer to Table 11 for examples of synthetic cannabinergic agonists. 

**Table 11:** Synthetic cannabinergic agonists.

	Synthetic Cannabinergic Agonists	Biological Effect(s) and/or Mechanism Of Action	Reference
i.	WIN55212-2 (Winthrop) - A mixed R/CB_2_R agonist. - Penetrates the CNS.	- Inhibits heroin-seeking behaviour in rats - Attenuates neurological damage and reduces infarct size in artery occlusion in rats - Reduction in glial damage after hypoxic-ischemic brain injury in preterm lambs - Antinociceptive activity in rat pain models	[6,58,136,389,390]
ii.	CP-55940 (Pfizer)	- Inhibits heroin-seeking behaviour in rats	[136]
iii.	URB-597 (aka KDS-4103) (targets FAAH)	- Anxiety, cannabis-dependence, and hyperalgesia - Anti-depression	[391] [6]
iv.	PF-04457845 (Pfizer—targets FAAH)	Pain disorders (including osteoarthritis)	[342]
v.	V158866 (Pfizer—targets FAAG)	Pain disorders (including osteoarthritis)	[6]

Drugs that inhibit the cellular uptake and/metabolism of cannabinoids such as fatty acid amide hydrolase (FAAH) and monoacylglycerol lipase (MAGL) [184,392] may have benefits against diseases/disorders such as cancer, anxiety, neuropathic path, and inflammatory bowel disease [393]. Examples of drugs that inhibit the cellular uptake and/metabolism of cannabinoids are listed in Table 12. 

**Table 12:** Drugs that inhibit the cellular uptake and/metabolism of cannabinoids such as inhibitors of fatty acid amide hydrolase (FAAH) and monoacylglycerol lipase (MAGL).

	Drugs That Inhibit the Cellular Uptake of Cannabinoids	Mechanism of Action	Reference
i.	CBD	Inhibition of FAAH	[394]
ii.	LY-2183240	Inhibition of FAAH	[395]
iii.	V-158866 (Vernalis)	Inhibition of FAAH	[396]
iv.	VER-156084 (Vernalis)	Inhibition of FAAH	[397,398]
v.	URB597 (KDS-4103, Kadmus Pharmaceuticals),	Inhibition of FAAH	[399,400]
vi.	PF750 and PF-655	Inhibition of FAAH	[393]

Examples of drugs that inhibit the deactivation of the ECS [136] or drugs that inhibit endocannabinoid metabolism [57] are listed in Table 13.

**Table 13:** Drugs that inhibit endocannabinoid metabolism and the deactivation of the ECS.

	Drugs That Inhibit the Deactivation	Biological Effect(s) and/or Mechanism of Action	Reference
i.	AM404	Blocks endocannabinoid transport	[136]
ii.	OMDM-8	Blocks endocannabinoid transport	[136]
iii.	AM1172 (University of Connecticut/University of California)	Blocks endocannabinoid transport	[136]
iv.	FAAH (fatty acid amide hydrolase)	Deactivates/degrades AEA	[136]
v.	MAGL (monoacylglycerol)	Deactivates/degrades 2-AG	[136]

## 10. Conclusions and Future Direction

In recent years, genetic and pharmacological manipulation of the ECS has gained significant interest in medicine, research, and drug discovery and development. The distribution of the components of the ECS system throughout the body, and the physiological/pathophysiological role of the ECS-signalling pathways in many diseases (and the dysregulation thereof), all offer promising opportunities for the development of novel cannabinergic, cannabimimetic, and cannabinoid-based drugs that genetically or pharmacologically modulate the ECS via inhibition of metabolic pathways and/or agonism or antagonism of the receptors of the ECS. This modulation results in the differential expression/activity of the components of the ECS—beneficial in the treatment number of diseases. Further studies are required to investigate the molecular mechanisms of action of the ECS-signalling pathways involved in the aforementioned diseases.

The ECS is a complex molecular/biological system of multiple components that also play roles in other systems and physiological processes outside of the ECS. Thus, when targeting and modulating the expression of the ECS components, scientists and drug developers should consider the consequences on other physiological systems, and if the disruption of one component or pathway of the ECS will result in unwanted consequences in other areas of the ECS, and possibly adverse side effects. 

The findings of this review suggest that there are multiple cannabinergic secondary metabolites of *C. sativa* L. that may have potential as lead compounds in the development of cannabinoid-based pharmaceuticals for a variety of diseases. These may include single-molecule drugs or whole-plant extracts. Such drugs have already demonstrated promise in palliative care. Now that potential lead compounds from *C. sativa* L. have been identified, there are several following steps in the drug development process that involve validation of this potential, pre-clinical research, synthesis of the lead compound into an optimal form for delivery into the body, and ultimately clinical research. Other factors, such as benefits, efficacies of these lead compounds, mechanisms of action, risks, adverse effects, drug interactions, toxicities, possible synergies between other compounds, and cellular responses to other cannabinergic, cannabimimetic, and/or cannabinoid-based therapeutic drugs and traditional, mainstay drugs such as chemotherapeutics, should also be investigated. 

US Food and Drug Administration (FDA)-approval of such cannabinoid-based pharmaceuticals and substantiated clinical decision-making are strictly dependent upon the elucidation of the aforementioned factors and the generation of more evidence-based data.

## Figures and Tables

**Table 1:** Components of the ECS and possible targets for the treatment of various diseases.

Endo-Cannabinoids (“Endogenous Cannabinoids”/ eCBs)	Enzymes		Receptors	Transport Proteins
	**Synthesizing**	**Degradative**		
- 2-AG [17] - AEA [17] - PEA [17] - OEA [17]	- DAGL (2-AG) [18] - NAPE-PLD (AEA) [19]	- FAAH (AEA) [19] - NAAA (AEA) [19] - ABHD6 and ABHD12 (2-AG) [18] - MAGL (2-AG) [18]	- CB_1_R/CB_2_R - (2-AG and AEA) - GPR18 [20] - GPR55 [21,22], - GPR119 [23], - TRPV1 (AEA) [24] - PPARγ [15]	- FABPs [25,26] - HSP70s [27] - Serum albumin [27] - FAAH-like AEA transporter (FLAT) [28] - AMT aka EMT [19,29,30].

**Table 2:** Synthetic cannabinoids and their therapeutic window for pain.

	Synthetic Cannabinoids	Therapeutic Window	References
1.	HU-308 and AM-124 (CB_2_R agonists)	Pain and inflammation	[6]
2.	Pyrimidinecarboxamide (and its derivatives) (CB_2_R modulators)	Acute, chronic, and inflammatory pain	[6]
3.	JWH-133 (intrathecal administration)	Reduction in post-operative hypersensitivity	[57]
4.	Peripherally restricted CB_1_R agonists	Chronic pain	[58]

**Table 3:** Uses and properties of cannabinoids for bowel disorders.

	Disorder/Property	Reference
1.	Inflammatory bowel diseases such as Chron’s disease, ulcerative colitis and irritable bowel syndrome	[212,213,214,215,216,217,218,219,220,221,222]
2.	Secretion and motility-related disorders	[223]
3.	Ant-secretory	[224]
4.	Digestive	[225]
5.	Appetite-stimulant	[225]
6.	Anti-flatulent	[225]
7.	Anti-spasmodic (for diarrhoea and colic)	[225]
8.	Antiparasitic (for internal and external worms)	[225]
9.	Gastric ulcers	[225]
10.	Gastric neuroses	[225]
11.	Gastralgia (indigestion)	[225]
12.	Dispepsia	[225]
13.	Diarrhoea	[212,226]
14.	Abdominal cramping	[226]
15.	Abdominal pain	[226]
16.	Loss of appetite	[227]
17.	Anorexia	[219]
18.	Anti-inflammatory	[212]
19.	Anti-emetic	[212]
20.	Analgesic	[212]

**Table 4:** Commonly used cannabinoid receptor ligands in cannabinoid research [303].

CB_1_R-Selective Ligands		CB_1_ R/CB_2_R Ligands	CB_2_R-Selective Ligands	
Agonist	Antagonist/ Inverse Agonists	Agonists	Antagonist/ Inverse Agonists	Agonist
- Methanandamide - Arachidonyl-2-chloroethylamide (ACEA) - Arachidonylcyclo- - propylamide (ACPA)	- SR141716A - AM251 - AM288	- Δ^9^-THC - HU-210 - CP55940 - R-(+)-WIN552112 - AEA - 2-AG	- SR144528 - AM630	- JWH-015 - JWH-133 - HU-308 - AM1241 - GW405833 - GW842166X - O-1966

Of note is that CB_1_R inverse agonists may have adverse effects [304].

## Data Availability

Data sharing is not applicable to this article. No new data were created or analysed in this study.

## Abbreviations

2-AG	2-arachidonoylglycerol
*AEA*	*N*-arachidonoyl ethanolamide
CB_1_R	Cannabinoid receptor type 1
CB_2_R	Cannabinoid receptor type 2
FDA	Food & Drug Administration
NSAIDs	Nonsteroidal anti-inflammatory drugs
Δ^9^-THC	Δ^9^-Tetrahydrocannabinol
Δ^9^-THCA	Δ^9^-tetrahydrocannabinolic acid
Δ^9^-THCV	Δ^9^-tetrahydrocannabivarin
AM251	*N*-(piperidin-1-yl)-5-(4-iodophenyl)-1-(2,4-dichlorophenyl)-4-methyl-1*H*-pyrazole-3-carboxamide
AM281	*N*-(morpholin-4-yl)-1-(2,4-dichlorophenyl)-5-(4-iodophenyl)-4-methyl-1*H*-pyrazole-3-carboxamide
AM630	6-iodo-2-methyl-1-[2-(4-morpholinyl)ethyl]-1*H*-indol-3-yl](4-methoxyphenyl)methanone
AM1241	(2-iodo-5-nitrophenyl)-[1-(1-methylpiperidin-2-ylmethyl)-1*H*-indol-3-yl]-methanone
*AT*	Anandamide transporter
ACPA	Arachidonylcyclopropylamide
Aβ	Beta-amyloid
*CB*	Cannabinoid
*CBD*	Cannabidiol
CBDL	Cannabinodiol
*CBC*	Cannabichromene
CBCV	Cannabichromevarin
*CBL*	Cannabicyclol
*CBE*	Cannabielson
*CBG*	Cannabigerol
CBGV	Cannabigerovarin
CBGM	Cannabigerol Monoethyl Ether
*CBN*	Cannabinol
*CBT*	Cannabitriol
*CBV*	Cannabivarin
*COX2*	cyclooxygenase subtype 2
CP55940	(−)-*cis*-3-[2-hydroxy-4-(1,1-dimethylheptyl)phenyl]-*trans*-4-(3-hydroxypropyl)cyclohexanol
*ERK*	Extracellular-regulated kinase
*FAAH*	Fatty acid amide hydrolase
*GI*	Gastrointestinal
*GCPR*	G-Coupled Protein Receptor
HU-210	(6a*R*)-*trans*-3-(1,1-dimethylheptyl)-6a,7,10,10a-tetrahydro-1-hydroxy-6,6-dimethyl-6*H*-dibenzo[b,d]pyran-9-methanol
JWH-015	(2-methyl-1-propyl-1*H*-indol-3-yl)-1-naphthalenylmethanone
JWH-133	3-(1,1-dimethylbutyl)-6,6,9-trimethyl-6α,7,10,10α-tetrahydro-6*H*-benzo[c]chromene
PPAR*γ*	Peroxisome proliferator-activated receptor γ
TRVP1	Transient receptor potential vanilloid type 1
*MAP*	Mitogen-activated protein kinase
*R*-(+)-WIN55212	(*R*)-(+)-[2,3-dihydro-5-methyl-3-(4-morpholinylmethyl)pyrrolo-[1,2,3-de]-1,4-benzoxazin-6-yl]-1-naphthalenylmethanone
SR141716A	*N*-(piperidin-1-yl)-5-(4-chlorophenyl)-1-(2,4-dichlorophenyl)-4-methyl-1*H*-pyrazole-3-carboxamide hydrochloride
SR144528	*N*-[(1S)-endo-1,3,3-trimethyl bicyclo [2.2.1] heptan-2-yl]-5-(4-chloro-3-methylphenyl)-1-(4-methylbenzyl)-pyrazole-3-carboxamide
DAGL	Diacylglycerol lipase
MAGL	Monoacylglycerol lipase
NAPE-PLD	*N*-acetyl-phosphatidyl-ethanolamine-hydrolyzing phospholipase D
PEA	Palmitoylethanolamide
OEA	Oleoylethanolamine
FAAH	Fatty acid amide hydrolase
NAAH	*N*-acylethanolamine acid amide hydrolase
ABHD6	Alpha/beta-Hydrolase domain containing 6
ABHD12	Alpha/beta-Hydrolase domain containing 12
GABA	Gamma aminobutyric acid
GPR55	G-protein coupled receptor 55
GPR18	G-protein coupled receptor 18
GPR119	G-protein coupled receptor 119
FABS	Fatty Acid Binding Protein
HSP70s	70 kilodalton heat shock proteins
AMT	Anandamide membrane transporter
EMT	Endocannabinoid membrane transporter
